# Viral Induction of Novel Somatic and Germline DNA Functions in Host Arthropods Opens a New Research Frontier in Biology

**DOI:** 10.3389/fmolb.2022.847670

**Published:** 2022-02-24

**Authors:** Timothy W. Flegel

**Affiliations:** Center of Excellence for Shrimp Molecular Biology and Biotechnology (Centex Shrimp) Faculty of Science, Mahidol University and National Center for Genetic Engineering and Biotechnology (BIOTEC), Bangkok, Thailand

**Keywords:** autonomous genetic modification, natural trasgenic organisms, viral copy DNA (vcDNA), heritable immunity, host reverse transcriptase, insects, crustaceans

## Introduction

At the outset, I would like to make it clear that the purpose of this article is to highlight the fundamental significance of recent discoveries that prove insects and crustaceans possess dedicated, natural, adaptive, antiviral mechanisms that involve production of DNA for somatic and hereditary functions. It is probable that these immune mechanisms do not occur in mammals and other vertebrates. Instead of clearing viruses as vertebrate survivors usually do, crustaceans and insects usually develop low level persistent infections (sometimes for multiple viruses) that can last for a lifetime but produce no gross signs of disease (Flegel, 2020). These persistent infections are not latent or chronic. They involve adaptive immunity that depends on shuttles between host and viral RNA and DNA rather than depending on antibodies (which crustaceans and insects do not produce). The hosts are persistently, actively infected and remain infectious for naïve members of their population, sometimes with lethal consequences.

This phenomenon of tolerating viruses in persistent infections has been called viral accommodation (Flegel, 2020). Based on accumulating evidence beginning around 2005 and on a previously underappreciated publication (Lin et al., 1999), it was hypothesized (Flegel, 2009) that the mechanism for viral accommodation involves natural, host autonomous genetic modification (AGMo) that gives rise to natural transgenic organisms (NTO). Briefly, it was proposed that crustaceans and insects can recognize foreign viral mRNA and make variable, fragmental cDNA copies (called viral copy DNA or vcDNA) from it using endogenous reverse transcriptase (RT). These copies are inserted into host genomic DNA by host integrase (IN). Some of these inserts subsequently produce antisense-RNA transcripts that can induce a protective, antiviral response via the RNA interference (RNAi) pathway. If they occur in germ-cell chromosomes, they are heritable, and may result in heritable, adaptive immunity.

The first insertion of non-retroviral, viral genome fragments into a host insect genome was described before 2000 (Lin et al., 1999), followed by others in both crustaceans and insects between 2006 and 2011 (Tang and Lightner, 2006; Maori et al., 2007; Saksmerprome et al., 2011). However, in 2012, after the discovery of such inserts in mammals, the term endogenous viral elements (EVE) was used to describe them and was subsequently generally adopted (Feschotte and Gilbert, 2012).

Now, several publications on insects have given experimental proof that AGMo is involved in the viral accommodation process in insects and that it gives rise to EVE resulting in NTO (Goic et al., 2013; Goic et al., 2016; Whitfield et al., 2017; Tassetto et al., 2019; Suzuki et al., 2020). They have also revealed that the EVE in mosquitos are located in PIWI-interacting RNA (PiRNA) gene-like clusters and produce anti-sense RNA that results in an RNAi response via a newly discovered type of PIWI protein. The insect work also revealed, unexpectedly, that the vcDNA was produced in both linear and circular forms and that it was capable of producing small interfering RNA (siRNA) as an immediate antiviral response (i.e., in addition to generating EVE that may lead to RNAi by a less-direct pathway) (Tassetto et al., 2017). These publications and those relating to crustaceans have recently been reviewed (Flegel, 2020; Taengchaiyaphum et al., 2021).

## Opinion

Here, I would like to focus on the fact that the insect discoveries negate the former, general belief that animals do not possess dedicated natural mechanisms for recording environmental adaptations in hereditary DNA (except perhaps for epigenetics). This expands the realm of AGMo and NTO well beyond that previously revealed in the archaebacteria and eubacteria that modify chromosomal DNA by the CRISPR mechanism for antiviral immunity (Mojica et al., 2005; Makarova et al., 2020). It also raises the possibility that similar mechanisms may occur in eukaryotes other than crustaceans and insects.

One good outcome of the revelation that AGMo is an ancient, natural biological process in bacteria and some animals is that the widespread public fear of genetically modified organisms (GMO) of any type may be unwarranted. Clearly, mankind has been living together with at least some NTO, growing them, eating them and using their products unknowingly for millennia without any apparent harm. It is also probable that NTO may include plants due to possible similar interactions with viral pathogens as those described for crustaceans and insects (da Fonseca et al., 2016).

With respect to shrimp and economic insects, the new knowledge raises tantalizing possibilities for future applications. For example, a clear understanding of the process of EVE generation, combined with the availability of specific pathogen free (SPF) breeding stocks should allow for the development of cultured crustaceans and insects such as bees and silkworms that can tolerate most or all their current viral threats. This should be achievable by injecting ovaries with appropriately-designed, vaccine-like reagents that would feed into the natural AGMo mechanism and result in acquisition of protective EVE at specifically designated positions in the genomic DNA of breeding-stock germ cells. Then, the subsequent offspring could be screened for presence of the resulting EVE. Use of chimeric primers for PCR would allow for detection and maintenance of the relevant EVE in the resulting NTO stocks, even in the absence of positive selective pressure from continuous exposure to the relevant viruses.

However, for me, the most important ramification of these discoveries is negation of the former paradigm that there were no dedicated, natural mechanisms in eukaryotes for recording adaptive, environmental responses in somatic or heritable, chromosomal DNA. This negation should open the way for fertile imaginations regarding other possible pathways that might have arisen during evolution as selected beneficial modifications of the underlying AGMo mechanisms to record adaptive environmental responses in DNA of animals and perhaps other eukaryotes. This might not be for germline issues only, but also for advantageous, non-heritable somatic processes. A good example of the latter is the use in host insects of vcDNA in linear and circular forms to not only generate EVE but also to launch an immediate, adaptive, systemic, anti-viral immune response (Tassetto et al., 2017). That response involves variable, individual somatic cell shuttles between RNA and DNA, and also involves exosome-like vesicles (ELV) that can serve as systemic transfer vehicles for siRNA and possibly vcDNA via insect and crustacean blood (called hemolymph).

Is it possible, if not probable, that evolution has morphed the currently known AGMo mechanisms to serve other purposes? What might be the possibilities in organisms such as plants and fungi, for example, that excel in asexual (somatic) reproduction? I also wonder, for example, whether the mechanisms might have morphed in a manner that has allowed DNA (amongst the most stable molecules we know) to serve as a somatic repository of long-term human memory. Key features of AGMo seem to be available for such a purpose. These would include the possibility of individual nerve cells to carry out autonomous genetic modifications to select or modify existing piRNA-like gene clusters or generate new piRNA like-genes via PIWI proteins. Some cells may also have the ability to transfer small RNA and DNA fragments (linear and/or circular?) among cells via exosome-like vesicles as insects do to defend against viruses. Indeed, exosomes carrying micro RNA (miRNA) are already known to occur in cerebrospinal fluid, but not in blood serum (Khasawneh et al., 2018; Akers et al., 2015). Currently, this miRNA is studied mostly in relationship to brain disfunctions.

With respect to being a candidate for involvement in long-term memory, a necessary but currently missing feature is some type of protein with RNA binding ability that might be activated by sensations (site, sound, touch, etc) to initiate a pathway leading to DNA-based long-term memory. If so, there is potential for enormous variation in miRNA among individual brain cells and even greater variation among individual people. As with EVE, these individual intercellular differences would be difficult to characterize, even by high throughput sequencing. Further, it is likely that the individual “specific DNA sequence” for a common sensory signal like “red” would vary from person to person and require communicative learning to achieve group consensus. Since extracellular products associated with these processes might be disruptive if distributed throughout our bodies, is it possible that the blood-brain barrier is there not just to keep some things out but also to keep some things in? Under the former paradigm, these thoughts would have been dismissed out of hand. Perhaps now, they might not be considered as “useless, wild imaginings.” This is just an example of the kind of speculation that I would like to engender in skilled specialists with active imaginations, and especially in the young who may be less encumbered by long held convictions that might limit their willingness to explore possibilities previously considered to be impossible.

The confirmation that NTO and its underlying mechanisms exist in at least some eukaryotes opens a fascinating new frontier. It is unpredictable where it may ultimately lead, but it has potential to fundamentally affect all the biological sciences. Thus, my main purpose here is to raise broad awareness of the underlying mechanisms of known AGMo that leads to NTO and to awaken the curiosity and fertile imaginations of those who may wish to explore this new frontier.
